# Predictive models of disease burden at diagnosis in persons with adult-onset ulcerative colitis using health administrative data

**DOI:** 10.1186/s12876-018-0924-6

**Published:** 2019-01-21

**Authors:** Sanjay K. Murthy, Tushar Shukla, Lilia Antonova, Marc-Andre Belair, Tim Ramsay, Zane Gallinger, Geoffrey C. Nguyen, Eric I. Benchimol

**Affiliations:** 1 University of Ottawa, The Ottawa Hospital, Ottawa Hospital Research Institute, IC/ES, 501 Smyth Road, Unit W1212, Ottawa, Ontario K1H 8L6 Canada; 2 The Ottawa Hospital, University of Ottawa, 451 Smyth Road, Ottawa, Ontario K1H 8L6 Canada; 3 Ottawa Hospital Research Institute, 737 Parkdale Ave, Room 67, Ottawa, Ontario K1Y 1J8 Canada; 4 IC/ES, Ottawa Hospital Research Institute, 1 Hospital Road, Whitehorse, Y1A 3H7 Yukon Canada; 5 University of Ottawa, Ottawa Hospital Research Institute, 501 Smyth Road, Room 1812F, Box 201, Ottawa, K1H 8L6 Canada; 6 Sinai Health System, University of Toronto, 600 University Ave, Toronto, ON M5G 1X5 Canada; 7 Sinai Health System, University of Toronto, Lunenfeld-Tanenbaum Research Institute, IC/ES Mount Sinai Hospital, 600 University Ave, Ste. 437, Toronto, Ontario M5G 1X5 Canada; 8 University of Ottawa, IC/ES, Children’s Hospital of Eastern Ontario (CHEO), CHEO Research Institute, Children’s Hospital of Eastern Ontario, 401 Smyth Rd, Ottawa, ON K1H 8L1 Canada

**Keywords:** Ulcerative colitis, Predictive modelling, Disease burden, Health administrative data

## Abstract

**Background:**

Health administrative data is increasingly used to conduct population-based health services research. A major limitation of these data for the study of inflammatory bowel diseases is the absence of detailed clinical information relating to disease burden. We used Ontario health administrative data to develop predictive models of disease burden at diagnosis in ulcerative colitis (UC) patients for future use in population-based studies of incident UC cohorts.

**Methods:**

Through chart review, we characterized macroscopic colitis activity and extent at diagnosis in consecutive adult-onset UC patients diagnosed at The Ottawa Hospital between 2001 and 2012. We linked this cohort to Ontario health administrative data to test the capacity of administrative variables to discriminate different levels of disease activity, disease extent and the disease burden (a composite of disease extent and activity). We modelled outcomes as binary (using logistic regression) and ordinal (using proportional odds regression) variables and performed bootstrap validation of our final models.

**Results:**

We tested 20 administrative variables in 587 eligible patients. The logistic model of total disease burden (severe and extensive colitis vs. all other phenotypes) showed moderate discriminatory capacity (optimism-corrected c-statistic value 0.729). Individual models of disease extent and disease activity showed poorer discriminatory capacity (c-statistic value < 0.7 for 3 of 4 models).

**Conclusions:**

Ontario health administrative data may reasonably discriminate levels of total disease burden at diagnosis in adult-onset UC patients. Our models should be externally validated before their widespread application in future population-based studies of incident UC cohorts to adjust for the confounding effects of differences in disease burden.

**Electronic supplementary material:**

The online version of this article (10.1186/s12876-018-0924-6) contains supplementary material, which is available to authorized users.

## Background

Inflammatory bowel diseases (IBD), comprising ulcerative colitis (UC) and Crohn’s disease (CD), afflict more than 0.3% of the population in developed countries and a growing number of persons in newly industrialized countries in Africa, Asia, and South America, contributing to an increasing worldwide burden of this disease [1]. IBD patients face significant morbidity from disease flares, hospitalizations and operations and have high per-patient direct and indirect costs [2, 3]. IBD care is becoming increasingly challenging for practitioners and costly for both patients and society due to the introduction of multiple expensive biologic therapies over the past 15 years [3, 4]. The mounting burden, complexity and costs of IBD care mandates intensive real-world investigation regarding disease epidemiology, prognostic factors and treatment effectiveness, in order to optimally target treatment strategies and health care resources.

Population-level health administrative data (HAD) is increasingly being used to conduct epidemiologic and health services research in patients with IBD. Such data offers a unique opportunity to study real-world health outcomes and determinants across large and diversified populations and a variety of practice settings. However, a significant limitation of using these data to study individuals with chronic diseases is the absence of prognostically-relevant clinical information relating to disease burden and disease course, which can confound observed associations and limit the ability to develop patient-specific management strategies.

In individuals with ulcerative colitis (UC), anatomic colitis extent and severity of colitis activity have been shown to be important predictors of disease prognosis and treatment response. Extensive colonic disease is a risk factor for poor treatment response [5, 6], aggressive disease behaviour[7] and colectomy [8–10], while severe colitis activity is associated with a sub-optimal response to medical therapy [6, 11–13] and a higher risk for colectomy [5, 11, 14–16]. Extensive [17–19] and severe [20, 21] colitis are also independent risk factors for developing colorectal cancer. The inability to stratify or control for these factors using health administrative data diminishes the validity and clinical applicability of population-based studies in UC patients.

To address these limitations, we sought to develop predictive models of disease burden at diagnosis that would accurately discriminate between prognostically-distinct sub-populations of UC patients, using demographic, clinical and health care utilization parameters available in Ontario health administrative data. Such models would allow investigators to better control for inherent differences in disease prognosis and study distinct UC populations over current methods in future population-based studies in incident UC cohorts.

## Methods

### Overview

We identified a reference cohort of consecutive newly-diagnosed UC patients at the Ottawa Hospital over a 11-year period, who we then characterized on macroscopic colitis extent and activity at diagnosis through chart review. We linked this cohort to provincial health administrative datasets in Ontario, Canada to analyze province-wide health care utilization and health outcomes in each individual, which would serve as predictive variables in our regression models. We developed parsimonious regression models of colitis extent, colitis activity and total colitis burden (combination of colitis extent and activity at diagnosis) through stepwise selection using administrative variables, comprising demographic, clinical and health services variables, at baseline and over 1 year following diagnosis (for time-dependent variables such as hospitalization and physician contacts). We performed bootstrap validation to derive robust performance measures for our models.

The study protocol and audit of patient medical records was approved by the Research Ethics Boards of the Ottawa Health Sciences Network (Ottawa, Canada). Individual patient consent was not required, as per the regulatory requirements of the Government of Canada for low-risk, chart review studies. The use of health administrative data in this project was authorized under section 45 of Ontario’s Personal Health Information Protection Act, which does not require review by a Research Ethics Board. All patient data was maintained confidential through storage in an encrypted password-protected file on a secure hospital server. Deterministic linkage of patient data to Ontario health administrative data was accomplished by linking health card number to an administrative data unique identification number. After linkage, all identifiable patient information was anonymized and encrypted. Following data linkage and acquisition of province-wide patient information necessary for model building, all data analyses were conducted by a programming analyst at the IC/ES under the guidance of study investigators.

### Study design, subjects and data sources

We first identified potential participants through the Ottawa Hospital Data Warehouse, a repository of hospitalizations, emergency department visits, day surgery visits (including endoscopy) and investigations (including laboratory data, pathology and diagnostic imaging) occurring at The Ottawa Hospital. The Ottawa Hospital is a tertiary care hospital and IBD referral center, serving a population of more than 1.2 million people across Eastern Ontario. We identified all adult patients (≥ 18 years old) with one or more hospital encounters associated with a diagnosis of Crohn’s disease (CD) (International Classification of Diseases (ICD), 9th Version (ICD-9) code 555.x (before April 1, 2002) or ICD 10th Version (ICD-10) code K50.x (after April 1, 2002)), UC (ICD-9 code 556.x or ICD-10 code K51.x), “noninfective gastroenteritis and colitis, unspecified” (ICD-10 code K52.9) or “indeterminate colitis” (ICD-10 code K52.3) and at least one lower endoscopic examination (flexible sigmoidoscopy or colonoscopy) between April 1, 2001 and March 31, 2012, as potential participants.

The medical records of these patients were manually reviewed by two study investigators (S.M. and T.S.) to identify patients that were diagnosed with adult-onset UC at The Ottawa Hospital based on clinical, endoscopic and histologic data. Physicians’ endoscopic reports were then reviewed to grade macroscopic colitis extent and severity of colitis activity at diagnosis. Colitis extent was classified as “extensive,” “left-sided” or “proctitis,” based on the Montreal Classification of IBD [22]. Colitis activity was graded as “severe,” “moderate” or “mild,” based on the criteria used to grade these categories in the Mayo endoscopic subscore [23]. We employed clinicians descriptors of visualized inflammation (i.e. “ulcers”, “friability”, etc.), to qualify disease activity by the Mayo endoscopic subscore wherever possible. If the full extent of colitis activity could not be discerned from an endoscopy record (such as in patients with extensive colitis who underwent only partial colonoscopy), computed tomographic (CT) scan results were supplemented to discern the full extent of disease. If a sufficiently detailed description of endoscopic findings needed to calculate the Mayo endoscopic subscore was not provided in a physician’s endoscopy note, the physician’s stated impression of colitis activity as “severe,” “moderate” or “mild” was used instead. Patients were excluded if either of colitis extent or activity could not be reasonably determined based on these criteria.

Using unique patient identifiers, we then linked these data at the patient level to multiple Ontario health administrative datasets, housed at the IC/ES [24], including datasets pertaining to physicians’ claims, hospitalizations, same day procedures, ambulatory care, and provincial registration. The goal of linkage to these datasets was to ascertain individual sociodemographic, clinical and health care utilization data from province-wide health care encounters, which could then be tested for their collective capacity to discriminate between discrete levels of disease burden at diagnosis. We tested variables that have either published evidence or reasonable face validity as measures of UC disease burden and that are accurately recorded in Ontario health administrative data. We tested health care utilization variables over 1, 2 and 3 years following diagnosis (for time-dependent variables such as hospitalization and physician contacts) for their association with outcomes of interest, retaining only the most useful definition of each of these variables in our final models. Individuals that did not have at least 3 years of continuous eligibility for health care coverage in Ontario following their UC diagnosis date, based on provincial registration data, were excluded. As health care in Ontario is funded under a single public payer system, capture of health care encounters within health administrative datasets is comprehensive for all legal long-term residents (> 99% of the population). Furthermore, demographic data and codes for procedures are accurately captured within Ontario health administrative data [25]. A description of the datasets and administrative codes used in this study are provided in Additional file 1: Table S1 and Additional file 2: Table S2.

We tested multiple administrative variables as candidate predictors in our regression models (Table 1). These predictors were chosen based on having reasonable construct validity for measuring UC disease extent and activity by three IBD experts (S.K.M., E.I.B. and G.C.N.). Predictive models were separately developed for disease activity, disease extent and total disease burden (a combination of disease extent [as measured by the Montreal classification of IBD]) and disease activity [as measured by the Mayo score], stratified at various levels for each measure (as listed in Tables 2 and 3).

**Table 1:** Administrative variables tested in multivariable models

1. Age at Diagnosis	
2. Sex	
3. Hospitalization for colitis within 30 days of diagnosis	
4. Hospitalization for colitis beyond 30 days following diagnosis^a^	
5. Number of hospitalizations for colitis beyond 30 days^a^	
6. Total number of hospitalization days for colitis^a^	
7. Non-hospitalization emergency department (ED) visit for colitis^a^	
8. Number of non-hospitalization ED visits for colitis^a^	
9. IBD-related physician encounter after diagnosis^a^	
10. Number of IBD-related physician encounters after diagnosis^a^	
11. IBD-related gastroenterologist (GI) encounter after diagnosis^a^	
12. Number of IBD-related GI encounters after diagnosis^a^	
13. IBD-related general surgeon (GS) encounter after diagnosis^a^	
14. Number of IBD-related GS encounters after diagnosis^a^	
15. Lower endoscopy after initial diagnostic endoscopy^a^	
16. Number of lower endoscopies after initial diagnostic endoscopy^a^	
17. Blood transfusion after diagnosis^a^	
18. Number of blood transfusion occurrences after diagnosis^a^	
19. Hospitalization associated with colitis-related complication (bowel perforation, peritonitis, peritoneal abscess, megacolon, venous thromboembolism or Clostridium difficile colitis)^a^	
20. IBD-related death or colectomy^a^	

^a^Variables assessed over 1-, 2-, and 3-year intervals following diagnosis – the definition that demonstrated the greatest bivariate association with an outcome was retained for testing in the multivariable model

**Table 2:** Performance of Logistic Regression Models

MODELS	Optimism-Corrected C-Statistic Values^a^	Unadjusted C-Statistic Values
*Colitis Extent*		
Model 1: Extensive Colitis (vs. Left-Sided Colitis/Proctitis)	0.695 (0.669,0.754)	0.710
Model 2: Extensive OR Left-Sided Colitis (vs. Proctitis)	0.716 (0.687,0.781)	0.737
*Colitis Activity*		
Model 3: Severe Colitis (vs. Mild-Moderate Colitis)	0.670 (0.637,0.733)	0.665
Model 4: Severe OR Moderate Colitis (vs. Mild Colitis)	0.663 (0.628,0.737)	0.675
*Total Disease Burden*		
Model 5: Severe AND Extensive Colitis (vs. all other categories)	0.729 (0.690,0.796)	0.734
Model 6: High Colitis Burden (vs. all other categories) ^b^	0.729 (0.702,0.789)	0.735

^a^C-statistic estimates and confidence intervals adjusted following bootstrap internal validation of regression models (1000 iterations)

^b^[severe AND extensive] OR [severe AND left-sided] OR [moderate AND extensive] **vs.** [severe AND proctitis] OR [moderate AND left-sided] OR [mild AND extensive] OR [moderate AND proctitis] OR [mild AND left-sided] OR [mild AND proctitis], based on the endoscopic mayo subscore of disease activity and Montreal classification of disease extent

**Table 3:** Discriminatory Capacity of Proportional Odds Regression Models

MODELS	Unadjusted C-Statistic Values
Model 7: Colitis Extent^a^	0.700 (0.664,0.739)
Model 8: Colitis Activity^b^	0.655 (0.617,0.699)
Model 9: Total Disease Burden^c^	0.706 (0.674,0.746)

^a^Extensive vs Left-sided vs Proctitis

^b^Severe vs Moderate vs Mild

^c^[severe AND extensive] OR [severe AND left-sided] OR [moderate AND extensive] vs. [severe AND proctitis] OR [moderate AND left-sided] OR [mild AND extensive] vs. [moderate AND proctitis] OR [mild AND left-sided] vs. [mild AND proctitis], based on the endoscopic mayo subscore of disease activity and Montreal classification of disease extent

### Analytic methods

Disease phenotypes were modelled both as binary variables, using multiple logistic regression (Models 1–6, Table 2), and as ordinal variables, using proportional odds regression (Models 7–9, Table 3), to determine if the modelling strategy impacted model performance. We used stepwise selection to derive parsimonious models. Variable entry into a model was permitted if its bivariate association with the outcome showed a *p*-value of < 0.2, and the variable was eliminated from the model if its p-value was ≥0.1 in the presence of other covariates (based on Type 3 Analysis of Effects). All candidate predictors were tested for multi-collinearity – if two or more variables showed a variance inflation factor > 10 or tolerance < 0.1, then the variable with the greatest bivariate association with the dependent variable was preferentially chosen. All time-varying parameters were ascertained over 1-, 2- and 3-year time periods following the date of endoscopic diagnosis, and the definition that showed the strongest bivariate association with each outcome was preferentially retained as the candidate variable definition for further testing in the regression model. Interaction terms were tested between final model variables.

Each of the final models underwent bootstrap internal validation to produce “optimism-corrected” estimates of model discriminatory capacity (based on the c-statistic value). The bootstrap method used simple random sampling with replacement for 1000 iterations, with each sample having the same size as the original cohort [26]. C-statistic values are considered reasonable if they exceed 0.7 and strong if they exceed 0.8 [27]. For the purpose of achieving finer discrimination between models, 0.7–0.79, 0.8–0.89 and > 0.9 were interpreted as having moderate, good and excellent discriminatory capacity, respectively. Calibration of the logistic regression models was assessed using the Hosmer-Lemeshow goodness-of-fit test; a non-significant *p*-value was interpreted as a well-calibrated model [28].

Multiple probability cut-points were further tested for each of the logistic regression models to determine the sensitivity, specificity, positive predictive value (PPV) and negative predictive value (NPV) of the models for classifying specific disease phenotypes. The preferred cut-point for each model was that which produced the highest value for PPV (with a minimum value of 0.8) at a sensitivity of ≥0.8 for the greater disease burden category in each model. These parameters would establish the utility of each model to identify subgroups of UC patients with greater disease burden for future studies using Ontario health administrative data.

All statistical analyses were conducted by using SAS Enterprise Guide 6.1 software (SAS Institute Inc., Cary, NC).

## Results

### Patients

A total of 587 newly-diagnosed UC patients were included in the study. The mean age of the cohort was 40 years (standard deviation [SD] 16.2 years)) and 296 (50.4%) were female. Of 581 patients with sufficient information on colitis extent, 255 (43.9%) had extensive disease, 209 (36.0%) had left-sided disease and 117 (20.1%) had proctitis. Of 541 patients with sufficient information regarding colitis activity, 168 (31.1%) had severe activity, 267 (49.3%) had moderate activity and 106 (19.6%) had mild activity. Roughly 20% of the cohort had severe AND extensive colitis while close to half of the cohort had “high disease burden” ([severe AND extensive] OR [severe AND left-sided] OR [moderate AND extensive] colitis).

### Logistic regression models (binary outcomes)

For all parameters relating to health care utilization and outcomes following diagnosis, the 1-year assessment window had at least as strong an association with measures of disease burden as the 2- and 3-year assessment windows. Therefore, health care utilization and outcomes occurring over 1-year were used to define these candidate predictors. Multicollinearity was not observed among any of the candidate predictors. The optimism-corrected and unadjusted c-statistic values for each of the six final logistic regression models are presented in Table 2. The parameter estimates and adjusted odds ratios for variables retained in each of the models are presented in Additional file 3: Table S3. Three of the models (Models 2, 5 and 6) displayed moderate discriminatory capacity, with the models of total colitis burden showing the greatest performance.

Sensitivity, specificity, PPV and NPV estimates for sequential ten percentile cut-points of predicted probability of having the greater disease burden are presented for each of the logistic regression models in Additional file 4: Table S4. Only two models met the minimum threshold for sensitivity and PPV (> 80% for each). At a probability threshold of 70% in Models 2 and 4, 84.3 and 80.5% of patients in the cohort could be predicted to have extensive/left-sided colitis (vs. proctitis) and moderate-to-severe colitis (vs. mild colitis), respectively, capturing 83.2% of individuals and 99.5% of patients with these phenotypes, respectively. Despite the reasonably high PPVs, specificity remained very poor at these thresholds (< 40% in Model 3 and < 1% in Model 4), in keeping with the fact that the prevalences of extensive/left-sided colitis and moderate-to-severe colitis were close to 80% in our cohort.

### Proportional odds regression models (ordinal outcomes)

C-statistic values for the three proportional odds models are presented in Table 3. The parameter estimates and adjusted odds ratios for variables retained in each of the models are presented in Additional file 5: Table S5. Two of the models displayed moderate discriminatory capacity.

## Discussion

Disease burden, extent and severity are important confounding factors in studies assessing outcomes and health care utilization in patients with IBD. In most cases, routinely collected health data do not contain these clinical variables, and therefore research relying on such data is frequently subject to bias. Combining administrative variables to predict disease phenotype may be one approach to limit confounding in future population-based studies in IBD patients that rely on administrative data.

We have conducted the first study to evaluate the utility of administrative data to discriminate different levels of UC disease burden at diagnosis. While administrative variables were somewhat modest at discriminating levels of colitis extent or activity in isolation, they performed considerably better at discriminating composite measures of disease burden that combined colitis extent and activity. This likely relates to the parameters that we chose to model outcomes in our study, such as future hospitalization, specialist care and complications, which are more likely to be impacted by the total magnitude of inflammatory burden than individual characteristics relating to colitis burden. Ultimately, predicting total colitis burden would be most relevant when adjusting for differences in inflammatory burden in future population-based studies of incident UC cohorts. Notably, we did not observe any improvement in model performance using proportional odds regression. Importantly, our models should be validated in other independent cohorts before their widespread application.

Model #5 (severe and extensive colitis vs. all other categories) has the greatest potential for utility in future studies, as it comprised just five health care utilization variables that can be accurately captured within health administrative data. Application of this model to an incident UC cohort derived using routinely collected data would generate a variable for predicted probability of having greater colitis burden at diagnosis, which could then be used as a matching or adjustment variable when evaluating exposure-outcomes associations. Coding macros could be created and adapted to the local administrative database structure to rapidly derive these predicted probabilities for repeated use. A notable limitation to the application of these models is that the exposure of interest could not be one of the variables in the models or act through one or more of the variables in the models. Furthermore, as the model variables included health care utilization and outcomes occurring during the one year priod following diagnosis, our models would only be suitable for the study of outcomes occurring at least one year after diagnosis. The fact that the 1-year definitions for model parameters were as strongly associated (or stronger) with measures of disease burden as the 2- and 3-year definitions suggests that health care utilization and adverse events are most prominent in the first year following UC diagnosis.

Of the candidate predictors, hospitalization for a colitis flare within 30 days following diagnosis, emergency department visit for a colitis flare, IBD-related physician visit and IBD-related visit to a general surgeon were most strongly and consistently predictive of greater disease burden across the models. This provides a window into the health care utilization patterns of UC patients in Ontario based on disease burden at diagnosis and may be useful for health care policy planning in this population. A study from the University of Manitoba similarly used health care utilization parameters to discriminate clinical disease course over one year among prevalent IBD cases [29]. Health care utilization has also been shown to accurately identify individuals with IBD and other chronic disease using health administrative data in Ontario and other jurisdictions [30–34].

Despite possessing reasonable discriminatory capacity for total colitis burden, administrative variables did not predict disease burden accurately enough to isolate subgroups of phenotypically-distinct UC patients for future study. We would recommend against adoption of any of the models for this purpose. While models # 2 and 4 showed a reasonably high PPV and sensitivity for extensive/left-sided colitis and moderate-to-severe colitis, respectively, the PPV may be overly optimistic in these models due to the greter prevalence of patients with higher levels of disease burden at diagnosis at our tertiary care center as compared to the general UC population. Furthermore, the specificity in both models was very low, indicating a high rate of false positives.

Our study has important limitations. We based our assessment of disease burden solely on endoscopic parameters, including macroscopic colitis extent and activity, as they have demonstrated prognostic value for important UC-related outcomes [35]. Clinicians also place a great deal of importance on endoscopic findings to guide management [36]. However, they may not capture the full breadth of disease burden among UC patients. Notably, other measures of UC disease burden that incorporate clinical signs and symptoms and laboratory parameters have also shown prognostic value [37]. Disease phenotype at diagnosis could also have been misclassified in some patients in our study due to its retrospective ascertainment. However, given the simplicity of the classification systems used for disease extent and activity (Montreal classification and endoscopic component of the Mayo score), and the availability of detailed endoscopy records, we have reasonable confidence in our ascertainment of these phenotypes. Some individuals could have also progressed to a more advanced disease phenotype over the course of a year, which could have influenced health care utilization and outcomes and reduced model performance. Notably, a recent meta-analysis of 30 studies reported a pooled rate of disease extension of just 17.8% over five years among UC patients with non-extensive disease at diagnosis [38].

Additionally, our ability to accurately assess health care utilization and disease outcomes over a one-year period following diagnosis depended on the completeness and accuracy of Ontario health administrative data. While major health care use in Ontario (such as hospitalizations and physician visits) would have been comprehensively captured, health care use outside of Ontario would not have been recorded. Procedural information is also very accurately coded within the Ontario hospital discharge abstract database and administrative codes for individual demographics and physician specialty are based on well-established and accurate data provided by the Ministry of Health and Long-Term Care of Ontario [25]. However, the quality of coding of major diagnoses (such as those used to construct the variable “hospitalization for colitis-related complications”) may have been considerably more variable within the discharge abstract database [25].

Additionally, while UC patients in the TOH cohort should be reasonably representative of the broader UC population socio-demographically, persons with greater disease burden may have been over-represented. Furthermore, health care utilization patterns, which were critical to model derivation, may vary across Ontario. Therefore, validation of our models in other jurisdictions is necessary before their widespread application.

## Conclusions

In summary, we have demonstrated that administrative variables can reasonably discriminate UC disease burden at diagnosis. Once validated, these models could be adopted to adjust for the confounding effects of disease burden in studies of incident UC cohorts using health administrative data.

## Additional files

Additional file 1: **Table S1.** Ontario Health Administrative Databases Used to Capture Study Information^£^. (DOCX 19 kb)

Additional file 2: **Table S2.** Administrative Codes Used to Ascertain Model Variables. (DOCX 18 kb)

Additional file 3: **Table S3.** Parameter Estimates and Odds Ratios for Logistic Regression Models of Disease Phenotype. (DOCX 17 kb)

Additional file 4: **Table S4.** Diagnostic Accuracy Measures for Each Tenth Percentile Probability Cut-Point in the Logistic Regression Models of Disease Phenotype. (DOCX 14 kb)

Additional file 5: **Table S5.** Parameter Estimates and Odds Ratios for Proportional Odds Regression Models of Disease Phenotype*. (DOCX 16 kb)
