# Supplementary material for: Predictive models of disease burden at diagnosis in persons with adult-onset ulcerative colitis using health administrative data
Source: BMC Gastroenterol. 2019 Jan 21;19:13. doi: 10.1186/s12876-018-0924-6 (PMC6341567; doi:10.1186/s12876-018-0924-6)
Supplement: Supplementary file 1 — Table S1. Ontario Health Administrative Databases Used to Capture Study Information£. (DOCX 19 kb) [file 12876_2018_924_MOESM1_ESM.docx]

| **Supplemental Table 3. Parameter Estimates and Odds Ratios for Logistic Regression Models of Disease Phenotype** | | | | | | |
| --- | --- | --- | --- | --- | --- | --- |
| **Variables Tested*** | **Model 1**  Parameter  Estimate  (95% CI)  Adjusted  Odds Ratio  (95% CI) | **Model 2**  Parameter  Estimate  (95% CI)  Adjusted  Odds Ratio  (95% CI) | **Model 3**  Parameter Estimate  (95% CI)  Adjusted  Odds Ratio  (95% CI) | **Model 4**  Parameter  Estimate  (95% CI)  Adjusted  Odds Ratio  (95% CI) | **Model 5**  Parameter  Estimate  (95% CI)  Adjusted  Odds Ratio  (95% CI) | **Model 6**  Parameter  Estimate  (95% CI)  Adjusted  Odds Ratio  (95% CI) |
| Intercept | -1.43  (-1.88,-0.98) | -1.25  (-1.49,-1.00) | 0.289  (-0.451,1.03) | 0.717  (0.306,1.13) | -2.76  (-3.53,-2.00) | -0.930  (-1.39,-0.470) |
| Age at UC Diagnosis  (per year) | -- | -- | 0.0127  (-.00143,0.0268)  1.01  (0.999,1.03) | -- | -- | -- |
| Female Sex | -- | -- | -0.795  (-1.24,-0.353)  0.452  (0.291,0.703) | -- | -- | -0.4711  (-0.847,-0.0948)  0.624  (0.429,0.909) |
| Hospitalization for colitis flare within 30 days of diagnosis? (Y/N) | 1.03  (0.291,1.78)  2.81  (1.34,5.90) | 0.657  (-0.00931,1.32)  1.93  (0.991,3.75) | -- | -- | 0.892  (0.196,1.59)  2.44  (1.21,4.90) | -- |
| Hospitalization for colitis flare beyond 30 days of diagnosis? (Y/N) | -- | -- | -- | -1.86  (-3.61,-0.110)  0.156  (0.027, 0.895) | -- | 1.17  (0.294,2.05)  3.23  (1.34,7.75) |
| Number of hospitalizations for colitis flare beyond 30 days following diagnosis | -- | -- | -- |  | -- | -- |
| Total number of days spent in hospital for colitis flare | -- | -- | -- | 0.266  (-0.00801,0.540)  1.30  (0.992,1.72) | -- | -- |
| Emergency department visit for colitis flare (without hospitalization)? (Y/N) | 0.965  (0.336,1.59)  2.63  (1.40,4.93) | 1.66  (0.572,2.76)  5.29  (1.77,15.8) | -- | 2.16  (0.354,3.96)  8.64  (1.43,52.4) | 0.669  (0.0415,1.30)  1.95  (1.04,3.66) | 2.70  (1.38,4.03)  14.9  (3.96,56.4) |
| Number of emergency department visits for colitis flare (without hospitalization) | -- | -0.777  (-1.48,-0.0764)  0.460  (0.228,0.926) | -- | -1.07  (-1.92,-0.212)  0.344  (0.146,0.809) | -- | -1.25  (-1.99,-0.498)  0.288  (0.136,0.607) |
| IBD-related physician encounter following diagnosis? (Y/N) | 0.974  (0.458,1.49)  2.65  (1.57,4.47) | -- | 0.851  (0.324,1.38)  2.34  (1.38,3.96) | 0.467  (-0.0291,0.963)  1.60  (0.971,2.62) | 1.01  (0.178,1.85)  2.76  (1.19,6.37) | 0.826  (0.311,1.34)  2.28  (1.37,3.82) |
| Number of IBD-related physician encounters following diagnosis | -- | 0.0227  (0.00377,0.0416)  1.02  (1.00,1.04) | 0.0716  (0.0300,0.113)  1.07  (1.03,1.12) | -- | -- | -- |
| IBD-related gastroenterologist encounter following diagnosis? (Y/N) | -- | -- | -- | -- | -- | -- |
| Number of IBD-related gastroenterologist encounters following diagnosis | 0.416  (0.011,0.072)  1.04  (1.01,1.08) | -- | -- | -- | 0.0239  (-0.00354,0.0513)  1.02  (1.00,1.05) | 0.028  (-0.00120,0.0572)  1.03  (1.00,1.06) |
| IBD-related general surgeon encounter following diagnosis? (Y/N) | 1.05  (0.363,1.74)  2.86  (1.44,5.69) | 0.936  (0.285,1.587)  2.55  (1.33,4.89) | -- | 1.17  (-0.147,2.49)  3.23  (0.863,12.1) | 1.13  (0.488,1.77)  3.09  (1.63,5.87) | 1.55  (0.673,2.43)  4.72  (1.96,11.4) |
| Number of IBD-related general surgeon encounters following diagnosis | -- | -- | -- | -- | -- | -- |
| Lower endoscopy following initial diagnostic endoscopy? (Y/N) | -- | -- | -- | 0.939  (0.338,1.54)  2.56  (1.40,4.66) | -- | -- |
| Number of lower endoscopies following initial diagnostic endoscopy | -0.283  (-0.552,-0.0138)  0.754  (0.576,0.986) | -- | -- | -- | -- | -- |
| Blood transfusion following diagnosis? (Y/N) | -- | -- | -- | -- | -- | 1.61  (-0.0788,3.30)  4.99  (0.92,27.0) |
| Number of times requiring blood transfusion following diagnosis | -- | -- | -- | -- | -- | -- |
| Colitis-related complication^Ω^ (Y/N) | -- | -- | -- | -- | -- | -2.76  (-4.89,-0.637)  0.063  (0.008,0.529) |
| Death or Colectomy related to IBD? (Y/N) | -- | -- | -- | -- | -- | -- |

* Age, sex and hospitalization within 30 days of diagnosis ascertained at time of diagnosis; all other variables ascertained over one year following diagnosis

^Ω^ Includes hospitalization for bowel perforation, peritonitis, megacolon, abdominal infection, sepsis, venous thromboembolism or C. difficile

Model 1: Extensive colitis vs. left-sided/procititis

Model 2: Severe vs. moderate/mild colitis

Model 3: Extensive/left-sided colitis procititis

Model 4: Severe/moderate vs. mild colitis

Model 5: Severe+extensive colitis vs. other

Model 6: High colitis burden (severe+extensive or severe+left-sided or moderate+extensive colitis) vs. other
