# Supplementary material for: Predictive models of disease burden at diagnosis in persons with adult-onset ulcerative colitis using health administrative data
Source: BMC Gastroenterol. 2019 Jan 21;19:13. doi: 10.1186/s12876-018-0924-6 (PMC6341567; doi:10.1186/s12876-018-0924-6)
Supplement: Supplementary file 2 — Table S2. Administrative Codes Used to Ascertain Model Variables. (DOCX 18 kb) [file 12876_2018_924_MOESM2_ESM.docx]

| **Supplemental Table 5. Parameter Estimates and Odds Ratios for**  **Proportional Odds Regression Models of Disease Phenotype*** | | | |
| --- | --- | --- | --- |
| **Variables Tested*** | **Model 7**  Parameter  Estimate  (95% CI)  Adjusted  Odds Ratio  (95% CI) | **Model 8**  Parameter  Estimate  (95% CI)  Adjusted  Odds Ratio  (95% CI) | **Model 9**  Parameter Estimate  (95% CI)  Adjusted  Odds Ratio  (95% CI) |
| Intercept 1  (category 1 vs. category 2/3) | -0.650  (-1.017,-0.283) | -0.994  (-1.25,-0.742) | -0.496  (-0.877,-0.114) |
| Intercept 2  (category 1/2 vs. category 3) | 1.202  (0.823,1.58) | 1.40  (1.14,1.67) | 0.842  (0.455,1.23) |
| Age at UC Diagnosis  (per year) | -- | -- | -- |
| Female Sex | 0.384  (0.0659,0.703)  1.47  (1.07,2.02) | -- | 0.502  (0.161,0.842)  1.65  (1.18,2.32) |
| Hospitalization for colitis flare within 30 days of diagnosis? (Y/N) | -1.06  (-1.77,-0.36)  0.345  (0.171,0.696) | -0.782  (-1.39,-0.172)  0.458  (0.249,0.842) | -1.17  (-1.99,-0.348)  0.311 (0.137,0.706) |
| Hospitalization for colitis flare beyond 30 days of diagnosis? (Y/N) | -- | -- | -- |
| Number of hospitalizations for colitis flare beyond 30 days following diagnosis | -- | -- | -- |
| Total number of days spent in hospital for colitis flare | -- | -- | -- |
| Emergency department visit for colitis flare (without hospitalization)? (Y/N) | -1.01  (-1.63,-0.387)  0.366  (0.197,0.679) | -1.46  (-2.46,-0.455)  0.233  (0.086,0.635) | -2.28  (-3.52,-1.04)  0.102  (0.030,0.353) |
| Number of emergency department visits for colitis flare (without hospitalization) | -- | 0.701 (0.114,1.29)  2.02  (1.12,3.63) | 0.876 (0.233,1.52)  2.39  (1.26,4.55) |
| IBD-related physician encounter following diagnosis? (Y/N) | -0.783  (-1.21,-0.355)  0.457  (0.298,0.702) | -- | -0.608  (-1.06,-0.157)  0.544  (0.347,0.854) |
| Number of IBD-related physician encounters following diagnosis | -0.0453  (-0.0802,-0.0104)  0.956  (0.923,0.990) | -0.153  (-0.230,-0.0766)  0.858  (0.795,0.926) | -0.0552  (-0.0952,-0.0152)  0.946  (0.909,0.985) |
| IBD-related gastroenterologist encounter following diagnosis? (Y/N) | -- | -- | -- |
| Number of IBD-related gastroenterologist encounters following diagnosis | -- | 0.126 (0.0410,0.211)  1.134  (1.042,1.235) | -- |
| IBD-related general surgeon encounter following diagnosis? (Y/N) | -0.786  (-1.47,-0.106)  0.455  (0.231,0.899) | -- | -1.373  (-2.26,-0.486)  0.253  (0.104,0.615) |
| Number of IBD-related general surgeon encounters following diagnosis | -- | -- | -- |
| Lower endoscopy following initial diagnostic endoscopy? (Y/N) | -- | -0.347  (-0.734,-0.0411)  0.707  (0.480,1.04) | -- |
| Number of lower endoscopies following initial diagnostic endoscopy | -- | -- | -- |
| Blood transfusion following diagnosis? (Y/N) | -- | -- | -1.45  (-3.09,0.194)  0.235  (0.045,1.21) |
| Number of times requiring blood transfusion following diagnosis | -- | -- | -- |
| Colitis-related complication^Ω^ (Y/N) | -- | -- | 2.46  (0.368,4.55)  11.7  (1.44,94.2) |
| Death or Colectomy related to IBD? (Y/N) | -- | -- | -- |

* Estimates are based on modelling less aggressive phenotype in each of the models

Model 7: Colitis extent (proctitis vs. left-sided vs. extensive)

Model 8: Colitis activity (mild vs. moderate vs. severe)

Model 9: Colitis burden ([moderate AND proctitis] OR [mild AND left-sided] OR [mild AND proctitis] vs. [severe AND proctitis] OR [moderate AND left-sided] OR [mild AND extensive] vs. [severe AND extensive] OR [severe AND left-sided] OR [moderate AND extensive])
