# Supplementary material for: Predictive models of disease burden at diagnosis in persons with adult-onset ulcerative colitis using health administrative data
Source: BMC Gastroenterol. 2019 Jan 21;19:13. doi: 10.1186/s12876-018-0924-6 (PMC6341567; doi:10.1186/s12876-018-0924-6)
Supplement: Supplementary file 3 — Table S3. Parameter Estimates and Odds Ratios for Logistic Regression Models of Disease Phenotype. (DOCX 17 kb) [file 12876_2018_924_MOESM3_ESM.docx]

| **Supplemental Table 4. Diagnostic Accuracy Measures for Each Tenth Percentile Probability Cut-Point**  **in the Logistic Regression Models of Disease Phenotype** | | | | | | | | | |
| --- | --- | --- | --- | --- | --- | --- | --- | --- | --- |
|  | | | | | | | | | |
|  | **Probability Cut-Points** | | | | | | | | |
|  | 10% | 20% | 30% | 40% | 50% | 60% | 70% | 80% | 90% |
| **Model 1: Extensive colitis vs. left-sided/procititis** | | | | | | | | | |
| Sensitivity | -- | 98.43% | 90.20% | 65.49% | 42.35% | 36.08% | 23.53% | 11.76% | 3.92% |
| Specificity | -- | 3.37% | 30.37% | 61.35% | 87.73% | 91.10% | 96.01% | 98.16% | 99.69% |
| PPV | -- | 44.35% | 50.33% | 57.00% | 72.97% | 76.03% | 82.19% | 83.33% | 90.91% |
| NPV | -- | 73.33% | 79.84% | 69.44% | 66.05% | 64.57% | 61.61% | 58.72% | 57.02% |
| **Model 2: Severe vs. moderate/mild colitis** | | | | | | | | | |
| Sensitivity | 100.00% | 91.07% | 50.00% | 35.12% | 21.43% | 15.48% | 12.50% | 8.33% | 1.79% |
| Specificity | 0.27% | 25.74% | 77.48% | 87.40% | 94.10% | 97.86% | 98.39% | 99.73% | 99.73% |
| PPV | 31.11% | 35.58% | 50.00% | 55.66% | 62.07% | 76.47% | 77.78% | 93.33% | 75.00% |
| NPV | 100.00% | 86.49% | 77.48% | 87.40% | 94.10% | 71.99% | 71.40% | 70.72% | 69.27% |
| **Model 3: Extensive/left-sided colitis procititis** | | | | | | | | | |
| Sensitivity | -- | -- | -- | -- | 97.20% | 93.97% | **83.19%** | 61.85% | 31.25% |
| Specificity | -- | -- | -- | -- | 9.40% | 24.79% | **38.46%** | 69.23% | 94.02% |
| PPV | -- | -- | -- | -- | 80.97% | 83.21% | **84.28%** | 88.85% | 95.39% |
| NPV | -- | -- | -- | -- | 45.83% | 50.88% | **36.59%** | 31.40% | 25.64% |
| **Model 4: Severe/moderate vs. mild colitis** | | | | | | | | | |
| Sensitivity | -- | -- | -- | -- | -- | 99.77% | **99.54%** | 49.43% | 22.76% |
| Specificity | -- | -- | -- | -- | -- | 0.00% | **0.94%** | 76.42% | 94.34% |
| PPV | -- | -- | -- | -- | -- | 80.37% | **80.48%** | 89.58% | 94.29% |
| NPV | -- | -- | -- | -- | -- | 0.00% | **33.33%** | 26.91% | 22.94% |
| **Model 5: Severe+extensive colitis vs. other** | | | | | | | | | |
| Sensitivity | 93.58% | 58.72% | 46.79% | 24.77% | 20.18% | 10.09% | 5.50% | 1.83% | 0.92% |
| Specificity | 21.18% | 78.84% | 87.32% | 92.49% | 96.24% | 98.59% | 99.53% | 100.00% | 100.00% |
| PPV | 24.00% | 41.29% | 48.57% | 45.76% | 57.89% | 64.71% | 75.00% | 100.00% | 100.00% |
| NPV | 93.64% | 88.16% | 87.32% | 82.77% | 82.49% | 81.08% | 80.46% | 79.92% | 79.78% |
| **Model 6: [Severe+extensive or severe+left-sided or moderate+extensive colitis] vs. other** | | | | | | | | | |
| Sensitivity | 100.00% | 96.96% | 89.35% | 76.43% | 56.27% | 41.83% | 32.32% | 22.81% | 9.51% |
| Specificity | 0.37% | 16.91% | 31.25% | 56.99% | 76.84% | 90.44% | 94.85% | 96.69% | 98.53% |
| PPV | 49.25% | 53.01% | 55.69% | 63.21% | 70.14% | 80.88% | 85.86% | 86.96% | 86.21% |
| NPV | 100.00% | 85.19% | 75.22% | 71.43% | 64.51% | 61.65% | 59.17% | 56.44% | 52.96% |

-- Valid estimates not attainable
