# Supplementary material for: Predictive models of disease burden at diagnosis in persons with adult-onset ulcerative colitis using health administrative data
Source: BMC Gastroenterol. 2019 Jan 21;19:13. doi: 10.1186/s12876-018-0924-6 (PMC6341567; doi:10.1186/s12876-018-0924-6)
Supplement: Supplementary file 4 — Table S4. Diagnostic Accuracy Measures for Each Tenth Percentile Probability Cut-Point in the Logistic Regression Models of Disease Phenotype. (DOCX 14 kb) [file 12876_2018_924_MOESM4_ESM.docx]

| **Supplemental** **Table 1:** Ontario Health Administrative Databases Used to Capture Study Information^£^ | | |
| --- | --- | --- |
| **Variables** | **Database** | **Database Description** |
| Hospitalizations, colitis-related complications, colectomies, blood transfusions, endoscopies | Canadian Institutes of Health Information (CIHI) Discharge Abstract Database (DAD) and Same Day Surgery database | Administrative, clinical (diagnostic and surgical), and demographic data on all hospital discharges and same day surgical (diagnostic or therapeutic) procedures in Ontario |
| Emergency department visits, blood transfusions | National Ambulatory Care Reporting System |  |
| Outpatient physician encounters, endoscopies | Ontario Health Insurance Plan (OHIP) | All Ontario physicians’ claims for diagnostic and procedural services |
| Age, sex and death date | Registered Persons Database (RPDB) | Registration data and vital statistics pertaining to all Ontario citizens |
| Ontario health care coverage | RPDB, OHIP, DAD | As above |
| ^£^ ICES data dictionary available at: https://datadictionary.ices.on.ca/Applications/DataDictionary/Default.aspx | | |

* http://www.ncbi.nlm.nih.gov/pubmed/24774473
