# Supplementary material for: Predictive models of disease burden at diagnosis in persons with adult-onset ulcerative colitis using health administrative data
Source: BMC Gastroenterol. 2019 Jan 21;19:13. doi: 10.1186/s12876-018-0924-6 (PMC6341567; doi:10.1186/s12876-018-0924-6)
Supplement: Supplementary file 5 — Table S5. Parameter Estimates and Odds Ratios for Proportional Odds Regression Models of Disease Phenotype*. (DOCX 16 kb) [file 12876_2018_924_MOESM5_ESM.docx]

| **Supplemental Table 2**. Administrative Codes Used to Ascertain Model Variables | |
| --- | --- |
| IBD-Associated Hospitalization | **DAD (Diagnostic Codes)**  Any hospitalization with a most responsible **or** co-morbid discharge diagnosis of ulcerative colitis (ICD-10 K51.x; ICD-9 556.x), Crohn’s disease (ICD-10 K50.x; ICD-9 555.x), indeterminate colitis (ICD-10 K52.3) or IBD unspecified (ICD-10 K52.9) |
| IBD-Associated Emergency Department Visit | **NACRS (Diagnostic Codes)**  Any ED visit diagnostic code for ulcerative colitis (ICD-10 K51.x; ICD-9 556.x), Crohn’s disease (ICD-10 K50.x; ICD-9 555.x), indeterminate colitis (ICD-10 K52.3) or IBD unspecified (ICD-10 K52.9) |
| IBD-Associated Outpatient Physician Encounter | **OHIP (Fee Codes):**  Any claim with a diagnostic code of 555 or 556 |
| Lower Endoscopy | **OHIP (Fee Codes):**  Z580 or Z555 +/- other codes  **DAD/SDS (Intervention Codes):**  ICD-9-CM (before 2002) - 45.21, 45.22, 45.23, 45.24, 45.25, 45.27  CCI (2002 onwards) - 2NK70, 2NK71, 2NM70, 2NM71, 2NQ70, 2NQ71, 1NK87, 1NM59, 1NM87, 1NQ59, 1NQ87, 1NQ89 |
| Partial or Total Colectomy | **OHIP (Fee Codes):**  S166, S167, S168, S169, S170, S171, S172, S173, S174, S188  **DAD/SDS (Intervention Codes):**  ICD-9-CM (before 2002)– 45.7x, 45.8x  CCI (2002 onwards) – 1NM87, 1NM89, 1NM91, 1NQ87, 1NQ89, 1NQ91 |
| Blood Transfusion | **DAD and NACRS**  Code BTANY |
| Megacolon | **DAD (Diagnostic Codes)**  ICD-10 K59.3; ICD-9 564.7 |
| Perforation of Intestine | **DAD (Diagnostic Codes)**  ICD-10 K63.1; ICD-9 569.83 |
| Peritonitis | **DAD (Diagnostic Codes)**  ICD-10 K65.0, K65.9; ICD-9 567.0, 567.2 |
| Peritoneal Abscess | **DAD (Diagnostic Codes)**  ICD-10 K65.1; ICD-9 567.22 |
| Venous Thromboembolism | **DAD (Diagnostic Codes)**  ICD-9 – 325.0, 437.6, 451.1 – 451.8, 452 (all), 453 (all), 415.1, 671.3 – 671.5, 671.9, V125.1  ICD-10 – G08, I80.1 – I80.3, I81, I82.x, I26.x O08.2, O08.7, O22.3, O22.5, O22.8, O22.9, O87.1, O87.3, O87.9, O88.2, I63.6, I67.6, K55.0 |
| Clostridium Difficile Colitis | **DAD (Diagnostic Codes)**  CD-10 A04.7; ICD-9 008.45 |

DAD = Discharge Abstract Database

NACRS = National Ambulatory Care Reporting System

OHIP = Ontario Health Insurance Plan

SDS = Same Day Surgery

ICD = International Classification of Diseases

CCI = Canadian Classification of Interventions
